# Alterations of the gut bacterial microbiota in rhesus macaques with SIV infection and on short- or long-term antiretroviral therapy

**DOI:** 10.1038/s41598-020-76145-8

**Published:** 2020-11-04

**Authors:** Summer Siddiqui, Duran Bao, Lara Doyle-Meyers, Jason Dufour, Yuntao Wu, Yao-Zhong Liu, Binhua Ling

**Affiliations:** 1 Tulane National Primate Research Center, Covington, LA 70433 USA; 2 Department of Biostatistics and Data Science, School of Public Health and Tropical Medicine, Tulane University, New Orleans, LA 70112 USA; 3 Department of Molecular and Microbiology, National Center for Biodefense and Infectious Diseases, George Mason University, Manassas, VA 20110 USA; 4 Tulane Center for Aging, School of Medicine, Tulane University, New Orleans, LA 70112 USA; 5 Department of Microbiology and Immunology, School of Medicine, Tulane University, New Orleans, LA 70112 USA; 6 Present Address: Texas Biomedical Research Institute, 8715 W Military Dr, San Antonio, TX 78227 USA

**Keywords:** Microbiology, Microbial communities, Microbiome

## Abstract

Gut dysbiosis and microbial translocation are associated with chronic systemic immune activation and inflammation in HIV-1 infection. However, the extent of restoration of gut microbiota in HIV-1 patients with short or long-term antiretroviral therapy (ART) is unclear. To understand the impact of ART on the gut microbiota, we used the rhesus macaque model of SIV infection to characterize and compare the gut microbial community upon SIV infection and during ART. We observed altered taxonomic compositions of gut microbiota communities upon SIV infection and at different time points of ART. SIV-infected animals showed decreased diversity of gut microbiome composition, while the ART group appeared to recover towards the diversity level of the healthy control. Animals undergoing ART for various lengths of time were observed to have differential gut bacterial abundance across different time points. In addition, increased blood lipopolysaccharide (LPS) levels during SIV infection were reduced to near normal upon ART, indicating that microbial translocation and immune activation can be improved during therapy. In conclusion, while short ART may be related to transient increase of certain pathogenic bacterial microbiome, ART may promote microbiome diversity compromised by SIV infection, improve the gut microbiota towards the healthy compositions and alleviate immune activation.

## Introduction

The human gut is home to a vast community of bacterial mutualist^1^. It plays an important role in rapid and long-term protection against pathogens. A normal gut microbiota harbors a composite and diverse ecosystem essential for immune homeostasis^2^. In HIV/SIV infection the gut-associated lymphoid tissues is the primary target for viral transmission, replication, and early CD4^+^ T cell depletion^3–5^. Rapid and massive destruction of gut mucosa after HIV/SIV infection is associated with depletion of CD4^+^ T cells and persistent immune activation^6,7^. The gastrointestinal (GI) barrier disruption leads to the dysfunction of gut homeostasis, which most likely contributes to chronic immune activation and translocation of bacterial products such as lipopolysaccharides (LPS), peptidoglycans and bacterial DNA into the circulatory system^8–11^.

Many diseases are associated with alterations in the composition of microbiome and GI inflammation^12–14^. Similarly, HIV/SIV infection causes significant changes in the gut’s microbiome. The microbial dysbiosis results in the overall loss of diversity with modifications to the major phyla Bacteroidetes, Firmicutes, and Proteobacteria^15^. The loss of beneficial bacterial genera, like Bacteroides, Lactobacillus, and Bifidobacterium, has been observed and associated with HIV-1 pathogenesis and plays an important role in HIV disease progression^16^. Moreover, the level of several pathogenic proteobacteria also increases during HIV infection^16–18^. Studies reported that the alterations in the gut microbiome and changes in dietary tryptophan catabolite by-product are associated with systemic immune activation, which also contributes to the disease progression^17,18^.

The large-scale metagenomic studies on the fecal bacterial composition suggest that intestinal microbiota diversity is based on many host-genetic and environmental factors. Variation in the gut microbiome at the genus and species level depends on the inter-individual difference, but the composition at the phylum level is relatively consistent among individuals^19^. In healthy adults, fecal biota consists of a vast majority of the gram-negative Bacteroidetes and gram-positive Firmicutes. However, the extent of changes of gut microbiota in HIV-1 patients with antiretroviral therapy (ART) is not completely understood and longitudinal dynamic changes are difficult to study partly due to practical challenges of human studies and many confounding factors such as smoking and other lifestyles practices.

The nonhuman primate (NHP) model of SIV infection and ART is instrumental in many aspects of HIV research. This model can control many confounding factors in HIV-1 infected humans, and the viral inoculation route and dosages are standardized. Although the composition of gut microbiota in macaques is slightly different from humans^6^, one could argue that observed changes of gut microbiomes from the NHP model, if occurred, can still provide important information related to the microbiome dynamics due to HIV/SIV infection and ART. Although there are accumulating studies on the NHP GI microbiome in healthy vs. SIV infection with or without ART^6,11,20–23^, longitudinal studies following animals under ART, as in our study, were rarely performed. Consequently, the effect of ART and duration of treatment on changes of the gut microbiome is unclear. Here, we used the rhesus macaque model of HIV infection to characterize the gut microbial community and compared microbiome composition in groups of healthy animals (NON_SIV), SIV-infected animals (SIV+), and SIV-infected animals receiving antiretroviral therapy (ART). Moreover, we investigated the effect of different lengths of ART on dynamic changes of the gut microbiota. We further examined the microbial translocation from the GI tract to the immune system and subsequent immune activation. Overall, our goal was to analyze the impact of ART on dynamic changes and restoration of normal gut microbiota upon SIV infection.

## Results

### Gut bacterial microbial diversity in SIV-infected rhesus macaques with or without antiretroviral therapy

We sequenced 16S rRNA V3 and V4 hypervariable regions of fecal DNA samples from all the animals, the sequence reads were within 300–500 nucleotides (nt) with a median length of 469 nt. Other shorter or longer fragments were excluded (Suppl. Fig. S1). We observed that changes occurred in the gut microbial community during SIV infection compared to healthy SIV-naive controls. We also found that the macaque gut microbiota had altered taxonomic composition between SIV+ and ART groups, as well as among different treatment periods, paralleling human studies. The microbiota from all the groups predominantly consisted of four predominant phyla: Firmicutes, Bacteroidetes, Spirochaetes, and Proteobacteria, which were accounted for > 90% of the total community in all the samples (Suppl. Table S1).

**Figure 1:**
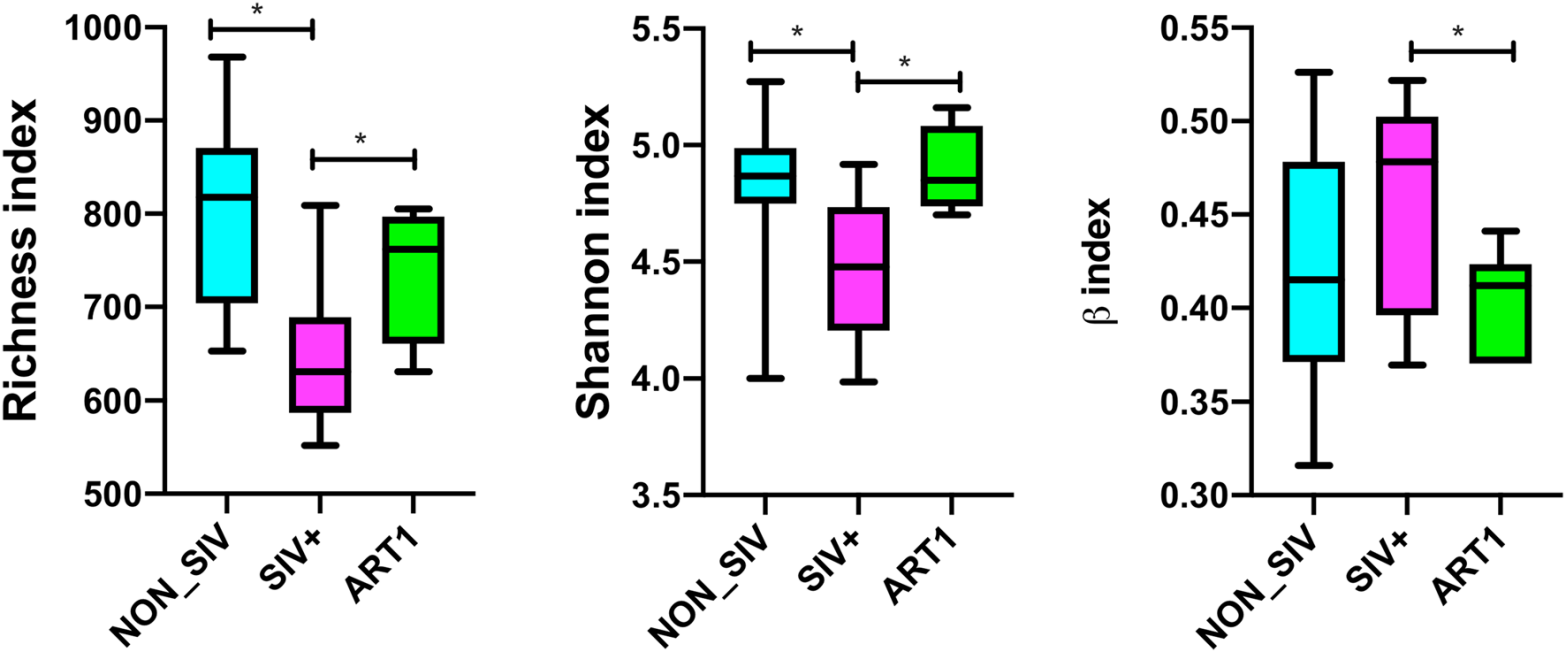
Microbial diversity between the control, SIV-infected and ART group. Alpha diversity is assessed and compared among three groups by (**A**) the richness index (*p* < 0.05) and (**B**) the Shannon index (*p* < 0.05). Beta diversity analysis of the fecal microbiota is assessed and compared among three groups by (**C**) (*p* < 0.05).

When sequences were condensed under 99% identity, a total of 9380 distinct operational taxonomic units (OTUs) were identified, based on which global diversity indices were calculated. To estimate the total number of OTUs in each data set, we used the Chao 1 estimator to assess the number of unseen OTUs present in the original sample. By the rarefaction analysis estimate, the increasing trend in species richness of bacterial communities within the ART group was comparable to that of NON_SIV controls. In contrast, richness of the SIV+ group was significantly reduced compared to groups of NON_SIV and ART treated animals (*p* < 0.05) (Fig. 1A). Richness indicates the number of species that contributes biodiversity. The SIV group appeared to have a reduced number of species (and hence compromised diversity) as compared with the NON_SIV controls, which appeared to be reversed by ART.

To compare the microbiome diversity between the three groups of NON_SIV, SIV+, and ART animals, we analyzed the α diversity, which is measured by species richness (Richness index) and evenness (Shannon index). The group of SIV+ animals had significantly decreased α diversity (*p* < 0.05) as compared to the NON_SIV control (Fig. 1A,B), while the diversity of the ART group appeared to recover to the levels of NON_SIV controls. This suggested that SIV infection may have decreased both richness and evenness of gut microbiota, while ART have reversed such trend.

In addition, to estimate the variation of microbial communities intra-group between different samples, β diversity analysis was performed based on both Bray–Curtis and Jaccard indices. The analysis revealed that compared to NON_SIV controls, SIV+ group had a higher β diversity (*p* < 0.05) (Fig. 1C), suggesting there’s a higher dissimilarity in intra-group community composition between different samples. Again, such a trend appeared to be reversed by ART (Fig. 1C), where β diversity of ART group was decreased as compared to that of SIV+, but comparable to the NON_SIV group.

### Differences in gut microbial composition in SIV-infected rhesus macaques with or without antiretroviral therapy

To understand the composition of fecal microbiota in each group, we conducted a taxon dependent analysis. This taxonomical analysis was performed at OTUs for NON_SIV, SIV+, and ART groups. Out of 137 core OTUs, 9 OTUs were identified at false discovery rate (FDR) < 0.10 for differential relative abundance among the three groups.

**Figure 2:**
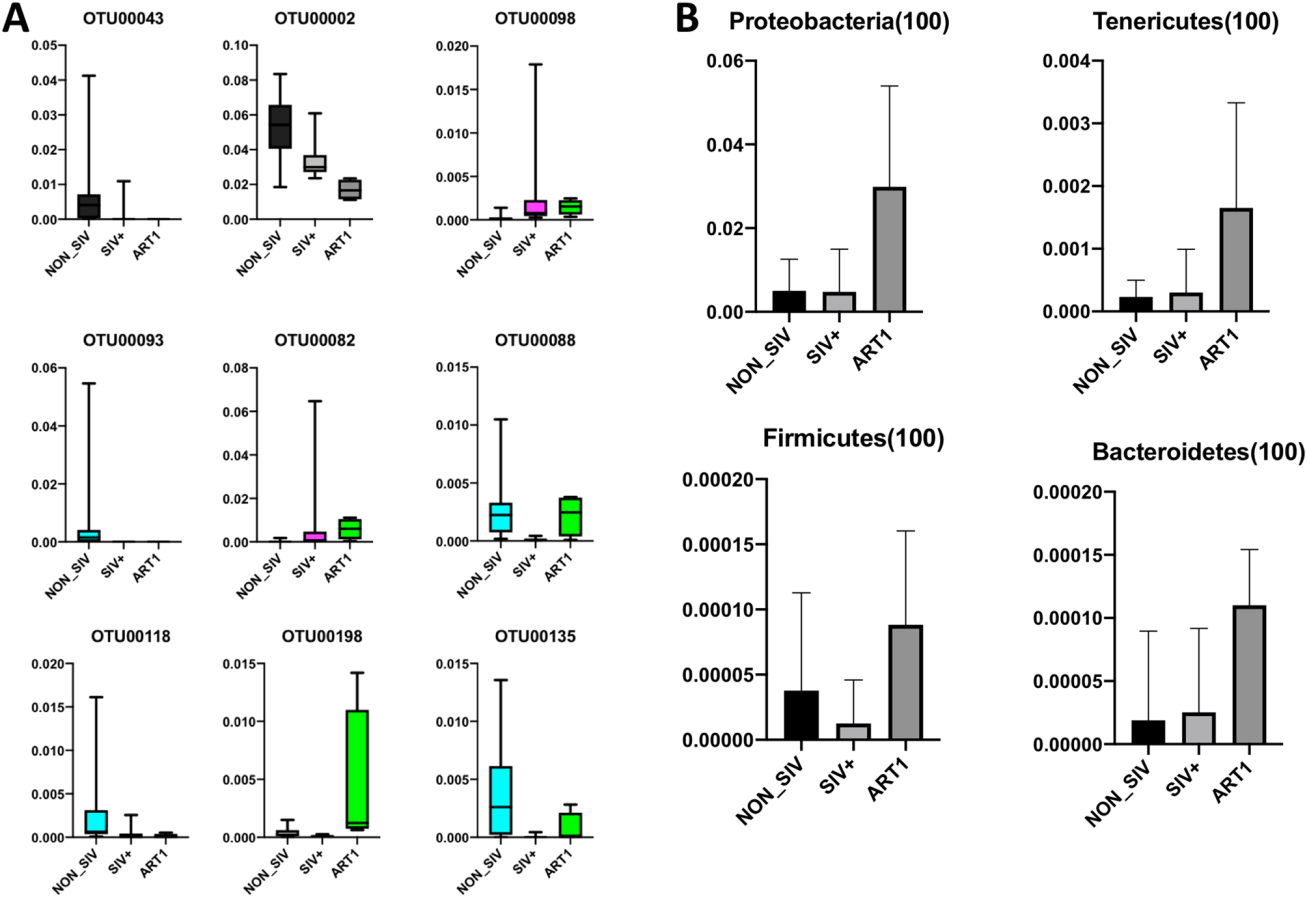
Identification of OTUs signatures between control, SIV-infected and ART treated group. The taxonomical analysis based on OTUs identified several OTUs (*p* < 0.001) (**A**) and phyla (*p* < 0.05) (**B**) with differential abundance between three groups.

**Figure 3:**
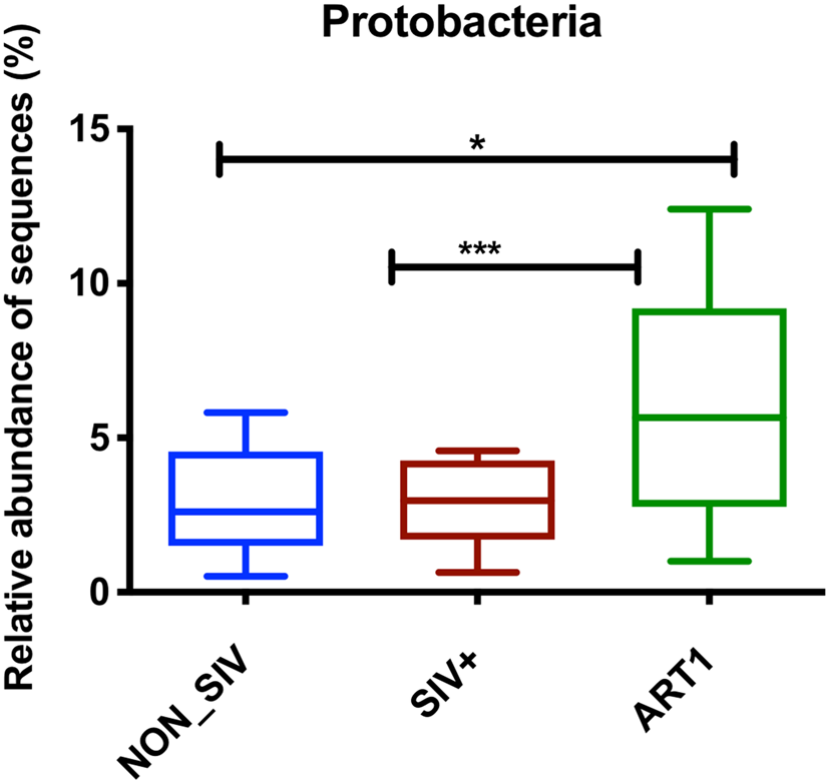
Relative abundance of Proteobacteria in ART treated group. The relative abundance analysis of the fecal microbiome at phylum level revealed that Proteobacteria were higher in ART treated group as compared to SIV+ (***p < 0.001) and NON_SIV (*p < 0.05). The relative abundance of Proteobacteria were compared between the SIV-infected and ART treated groups using Turkey’s multiple comparison test with 95% confidence intervals (CIs) and *p* value was adjusted by multiple comparisons and statistical difference among groups was estimated using ANOVA.

The most predominant genera in both NON_SIV and SIV+ groups were *Lachnospiraceae_unclassified* (OTU 00043), *Ruminococcaceae_unclassified* (OTU 00002) and (OTU 00118), *Lactobacillaceae* (OTU 00098) and (OTU 00082), *Bacteroidetes_unclassified* (OTU 00093), *Bacteroidales_unclassifies* (OTU 00088), and *Bacteria_unclassified* (OTU 00198) and (OTU 00135) (Fig. 2A). These OTUs predominantly belonged to bacterial phyla, which were *Bacteria_unclassified*, *Firmicutes*, and *Bacteroidetes* that accounted for > 97% of the total sequences. We observed that 4 phyla *Proteobacteria*, *Tenericutes, Firmicutes,* and *Bacteroidetes* were more abundant in the ART group (Fig. 2B). We also observed a statistically significant increase in the relative abundance of *Proteobacteria* in the ART group (Fig. 3) in comparison with NON_SIV and SIV+ groups. However, *Bacteroidetes* and *Firmicutes* did not change following ART (data not shown).

### Differentially abundant bacterial taxa in SIV-infected rhesus macaques with or without ART

**Figure 4:**
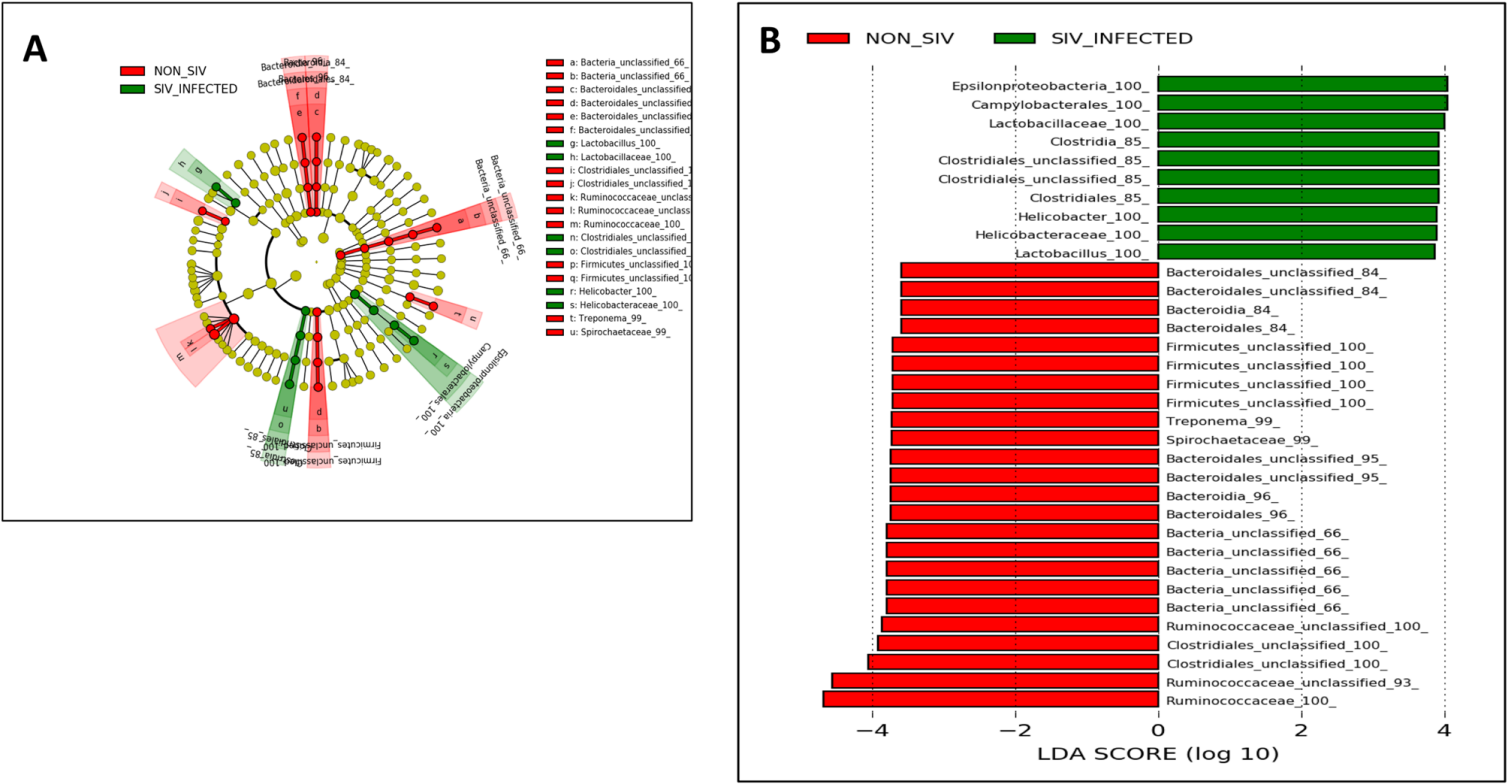
Taxonomic Cladogram obtained using LEfSe analysis of 16S rRNA sequences. LEfSe identified the taxa with the greatest differences in abundance between NON_SIV controls and SIV-infected animals. Taxa more abundant in NON_SIV group were shown in red; Taxa more abundant in SIV-infected group were shown in green. The color intensity of each dot was proportional to the effect size (**A**). The taxa more abundant in SIV-infected group were indicated with a positive LDA score (in green) and the taxa more abundant in NON_SIV group a negative score (in red). Only those taxa that reached a significant LDA threshold value of > 2 or < − 2 were shown (**B**). Statistical analyses were performed using the LEfSe, (https://huttenhower.sph.harvard.edu/lefse/).

To identify differences in the abundance of bacterial taxa, we used the linear discriminative analysis (LDA) effect size (LEfSe) biomarker discovery tool^24^. In comparison to the NON_SIV controls and SIV+ animals, there was a distinct difference revealed in a cladogram that represented the structure of the fecal microbiota and the predominant bacteria (Fig. 4A). The LEfSe analysis revealed 34 discriminative features (LDA score > 2, Fig. 4B). Based on LDA score analysis, we identified bacterial families differentially abundant among NON_SIV and SIV+ groups. Several members of *Bacteroidetes*, *Clostridial,* and *Ruminococcaceae* appeared more abundantly in the NON_SIV group and appeared to be beneficial or protective.

**Figure 5:**
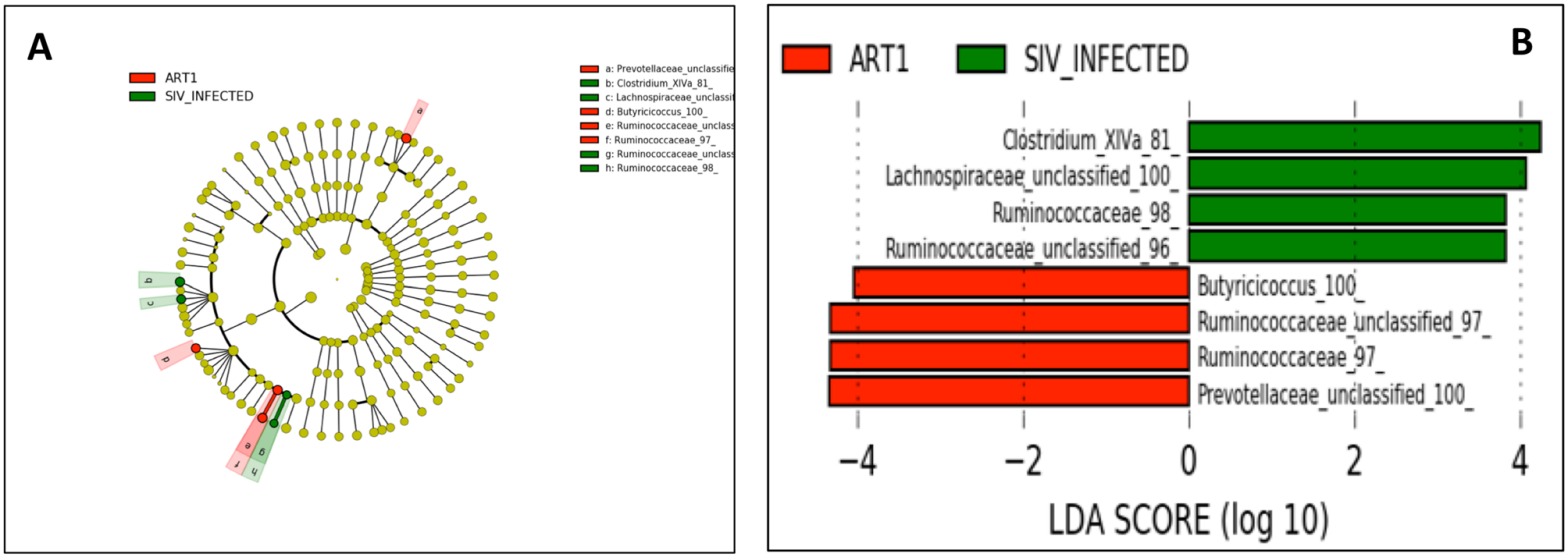
Taxonomic differences between the fecal microbiota of ART treated and untreated SIV-infected groups. LEfSe identified the taxa with the greatest differences in abundance between SIV-infected and ART treated animals. Taxa more abundant in SIV-infected group were shown in green; Taxa more abundant in ART treated group were shown in red. The color intensity of each dot was proportional to the effect size (**A**). The taxa more abundant in SIV-infected group were indicated with a positive LDA score (in green) and the taxa more abundant in ART treated group a negative score (in red). Only those taxa that reached a significant LDA threshold value of > 2 or < − 2 were shown (**B**). Statistical analyses were performed using the LEfSe, (https://huttenhower.sph.harvard.edu/lefse/).

Moreover, according to the LEfSe analysis, the discriminative microbial signature in the fecal microbiota was different between ART and SIV+ groups (Fig. 5A). Specifically, *Butyricicoccus, Ruminococcaceae, and Prevotellaceae* were more abundant in the ART group than the SIV+ group, whereas *Lachnospiraceae* and *Clostridium XIVa* were less abundant in the ART group (Fig. 5B). Of note, ART appeared to have recovered abundance *Ruminococcaceae*, a protective bacterial family that is low in abundance in SIV+ compared to NON_SIV.

### Changes in gut bacterial microbiome at different taxonomic levels

**Figure 6:**
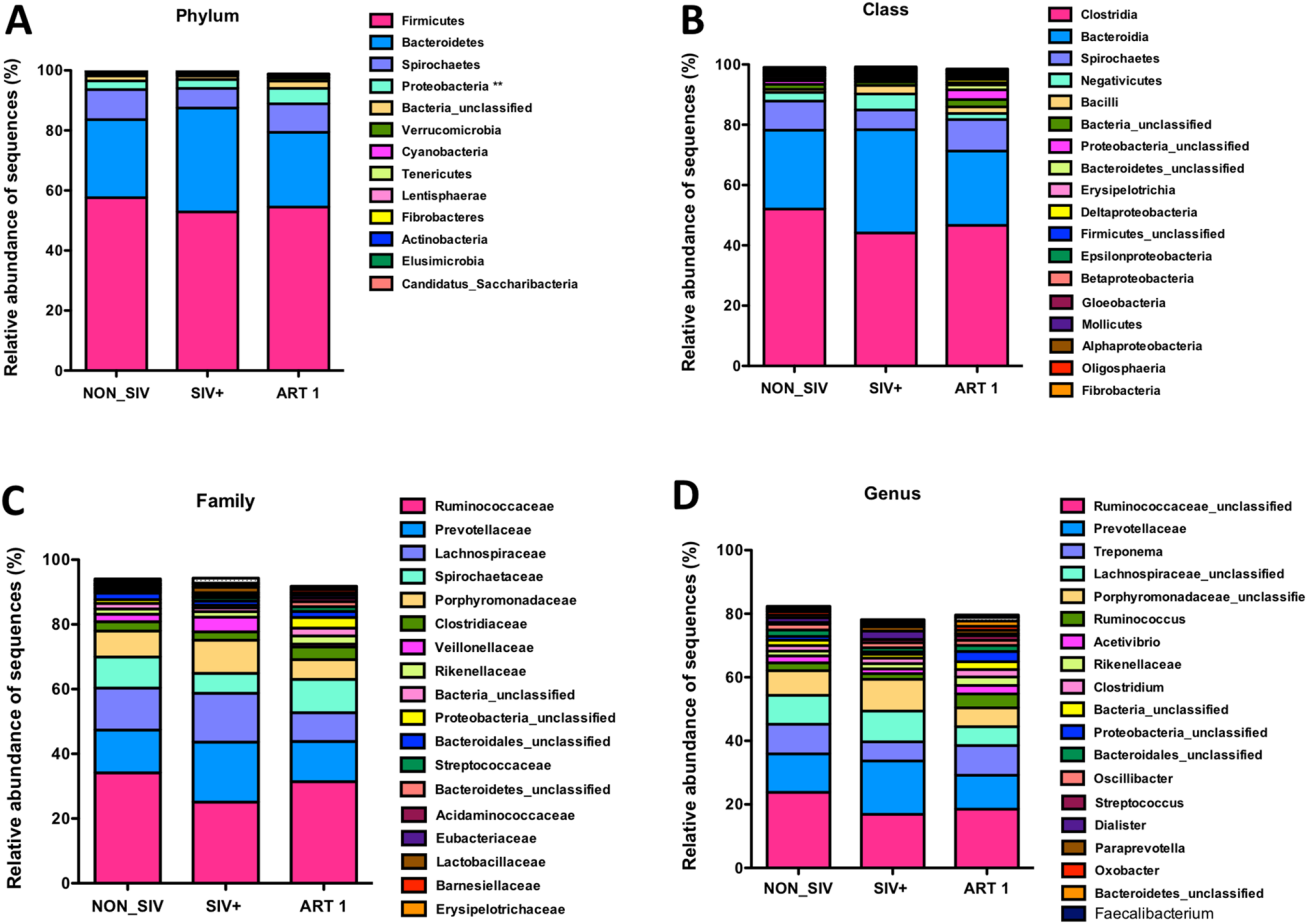
Gut microbiome relative abundance analysis in healthy controls, SIV-infected and ART treated animals. Comparisons of the relative abundance at the level of bacterial phylum (**A**), class (**B**), family (**C**), and genus (**D**) between control, treated and SIV infected groups were shown. There was an increase in abundance of Proteobacteria in the ART1 group than other two groups (**p < 0.01). At the class level, *Negativicutes* and *Bacilli* abundance were lower in ART1 as compared with SIV (NS). At family level the abundance of *Ruminococcaceae* (non-pathogenic bacteria) was lower in SIV but higher in ART1 as compared to NON_SIV (NS). At the genus level, *Prevotellaceae, Lachnospiraceae*_unclassified, and *Porphromonadaceae_unclassified* were more abundant in SIV group compared to ART1 group. Statistical analyses were performed using the GraphPad Prism [version 8.4.3(471); GraphPad Software. LLC, San Diego, CA, USA; https://www.graphpad.com/company/].

To understand the impact of SIV infection on gut microbiota and whether ART therapy can restore/reverse the effects at all taxonomic levels, we first compared gut microbiome relative abundance at the phylum level. The overall gut microbiome was dominated by the phyla Firmicutes and Bacteroidetes with a lower abundance of Proteobacteria. Although there was no statistically significant difference in the abundance of Firmicutes and Bacteroidetes among the three groups (NON_SIV, SIV+, ART), Proteobacteria was significantly more abundant in the ART group (*p* < 0.01) (Fig. 6A).

At the class level, *Bacilli* and *Negativicutes* had higher relative abundance in the SIV+ group (Fig. 6B), whereas *Clostridia, Spirochaetes,* and *Proteobacteria* were more prevalent in NON_SIV and ART group. Of the families, *Prevotellaceae*, *Lachnospiraceae_unclassified*, *Porphyromonadaceae_unclassified*, *Veillonellaceae* were more prevalent in the SIV+ group, whereas *Ruminococcaceae*, *Spirochaetaceae*, *Clostridiaceae,* and *Rikenellaceae* were more enriched in the ART group (Fig. 6C).

Further, the abundance of several genera was different in the SIV+ group compared with the ART group. *Prevotellaceae*, *Lachnospiraceae_unclassified*, and *Porphromonadaceae_unclassified* were more abundant in the SIV+ group, whereas the abundance of *Ruminococcaceae_unclassified*, *Treponema*, *Ruminococcus*, *Acetivibrio*, *Rikenellaceae*, and *Clostridium, Bacteroidales_unclassified* were higher in the ART group (Fig. 6D).

### Dynamic changes of gut microbial composition due to ART with different lengths of therapy

To assess the effect of treatment lengths on dynamic changes in the gut microbiome during different periods of antiretroviral therapy, we monitored four animals receiving ART at three different time points, which were ART1 (2 months of therapy), ART2 (6 months of therapy), and ART3 (9 months of therapy). The longitudinal analysis of 16S rRNA sequencing of stool samples collected at these three time points showed the distinct changes of the bacterial community, indicating the impact of length of ART on gut microbiome composition. The longitudinal analysis revealed several OTUs with differential abundance in the three groups of samples (Suppl. Table S1).

**Figure 7:**
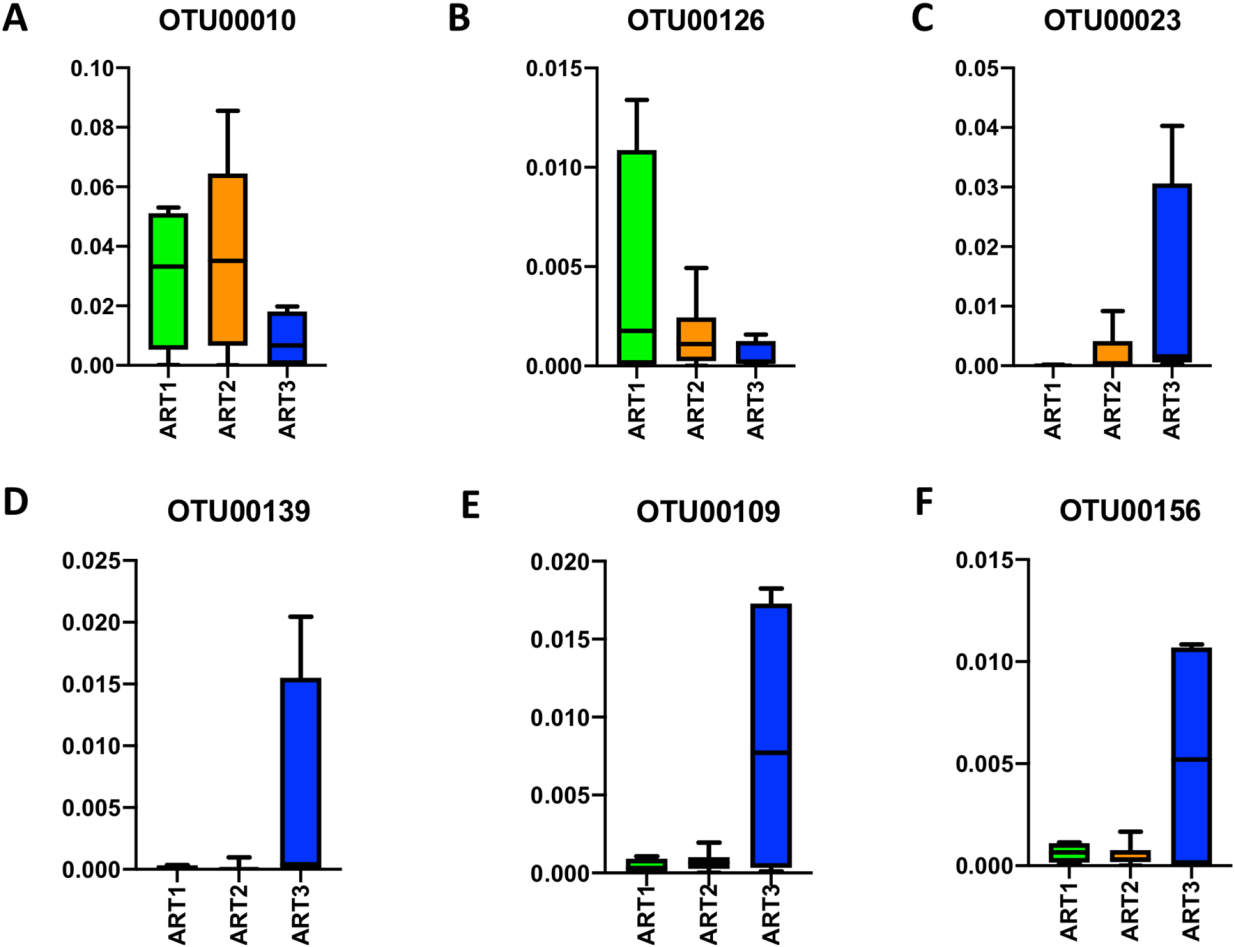
Identification of an OTU signature associated with ART treated group. The OTUs from phylum Proteobacteria were compared among three ART groups. The Y-axis represented the relative abundance. The abundance of Proteobacteria_unclassified (OTU00010) (*p* < 0.05) (**A**), *Helicobacter* (OTU00126) (*p* < 0.001) (**B**) decreased after the ART. The abundance of *Clostridium XIVa* (OTU00023) (*p* < 0.001) (**C**), *Clostridiales_unclassified* (OTU00139) (*p* < 0.001) (**D**), *Coprococcus* (OTU00109) (*p* < 0.001) (**E**), and *Ruminococcus* (OTU00156) (*p* < 0.05) (**F**) showed no or moderate increase at ART2 and marked increase at ART3. Statistical analyses were performed using the GraphPad Prism [version 8.4.3(471); GraphPad Software. LLC, San Diego, CA, USA; https://www.graphpad.com/company/].

As described above, the overall abundance of *Proteobacteria* was relatively increased in the ART1 group shown in Fig. 6A–D. Of note, it was decreased at 9 months of ART (ART3) (Fig. 7A). Interestingly, at the level of genera, ART was able to reduce the abundance of *Helicobacter* with 6 or 9 months of treatment (Fig. 7B).

Additionally, the relative richness of *Lachnospiraceae* family, particularly *Clostridium XIVa* (OTU00023) and the *Clostridiales_unclassified* (OTU00139) genus belonging to the phylum Firmicutes, remained at low levels during the ART1 and ART2 but reached a high level at ART3 (Fig. 7C,D). These results were in agreement with our LDA analysis result, where SIV infection group showed a higher level of abundance of the *Clostridium XIVa* than the ART1 group (Fig. 5B), suggesting a decreased level of *Clostridium XIVa* upon a short treatment (ART1). However, it appeared that a 9-month long treatment (ART3) may promote the increase of *Clostridium XIVa,* which rebounded to a higher level (Fig. 7C). In addition, the genera *Coprococcus* (OTU00109) and *Ruminococcus* (OTU00156) also increased in abundance as ART continues for a longer period of therapy (ART3) (Fig. 7E,F).

### Association of the microbial translocation marker between SIV infected, ART, and healthy controls

**Figure 8:**
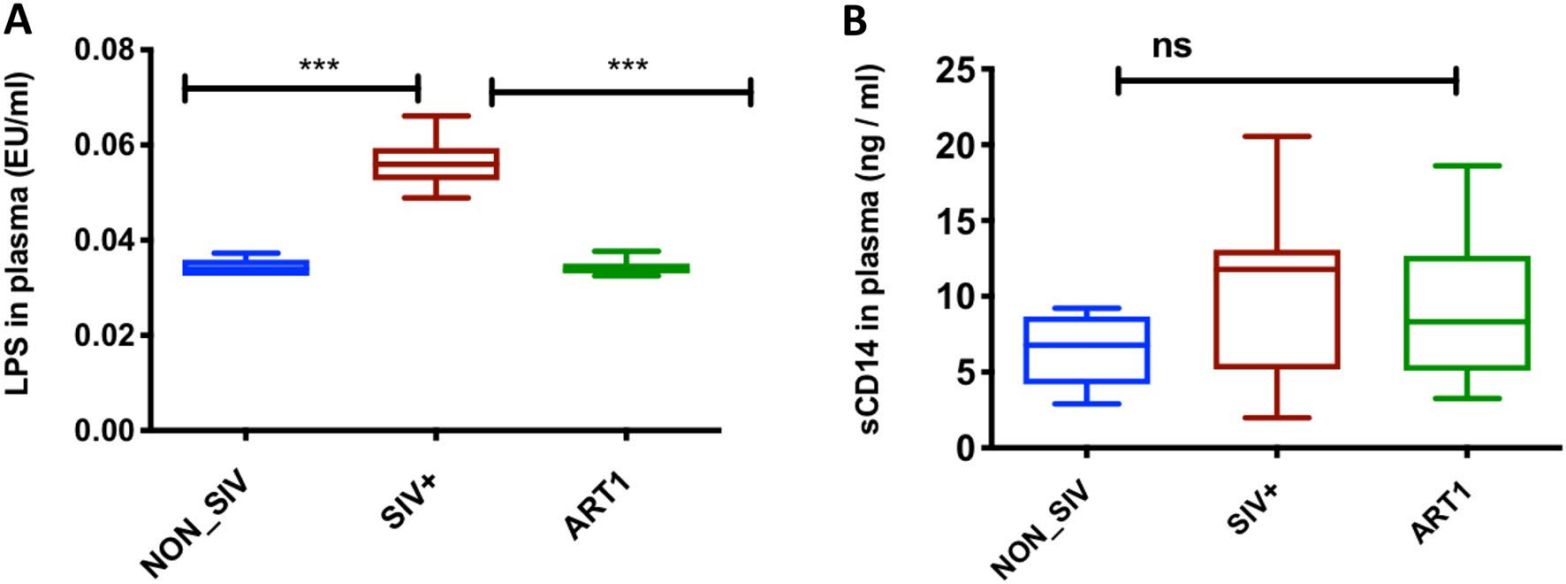
Plasma levels of LPS and sCD14 as microbial translocation marker. Plasma LPS levels were detected by limulus amoebocyte assay. The significant difference was observed in LPS levels between healthy controls and infected RMS (***p < 0.001) (**A**). There was no significant difference in sCD14 plasma levels but SIV infected group had slightly increased levels as compared to healthy control group (**B**). Statistical analyses were performed using the GraphPad Prism [version 8.4.3(471); GraphPad Software. LLC, San Diego, CA, USA; https://www.graphpad.com/company/].

The plasma LPS-binding protein and sCD14 levels were evaluated to examine the association between microbial translocation and SIV-mediated systemic immune activation and gastrointestinal leakage. In plasma, LPS levels were significantly higher in SIV+ group (0.056 ± 0.0054) than in NON_SIV group (0.034 ± 0.002), and were significantly decreased after ART (0.034 ± 0.002) (*p* = 0.001) (Fig. 8A). Plasma levels of sCD14 showed a trend of increase in SIV+ group (10.86 ± 5.994) in comparison with NON_SIV (6.47 ± 2.54) and ART groups (8.89 ± 4.30) but did not reach significant difference (Fig. 8B).

## Discussion

The analysis of changes in gut microbiota is very important in the context of HIV and SIV infection. The gut microbiota changes induced by HIV/SIV infection are linked to disruption of gut microbial composition and produce inflammation in gut mucosa^8,25,26^. Specifically, gut dysbiosis destroys the gut integrity barrier, enhances bacterial translocation of intestinal products from the lumen into the lamina propria, and increases immune activation^27–30^.

Normally human gut microbiome in a healthy individual is relatively stable over time^31^. However, gut dysbiosis occurs with enrichment or depletion of specific taxa during HIV^2,16,23,24^ or SIV infection^10,25^. In this study, we used a rhesus macaque model of HIV infection to determine changes in gut bacterial microbiomes upon SIV-infection and longitudinal changes at different time points of ART. We observed a decrease in α diversity in SIV-infected animals, in agreement with findings in the gut mucosa in HIV-1 infected patients^32^. Of note, this change does not occur in acute SIV infection^6^. Interestingly, reflected by Shannon and Richness indices, 2 months of antiretroviral therapy (ART1) significantly improved the gut microbial communities with greater richness, evenness, and between-animal homogeneity in terms of bacterial OTUs compared with SIV infection without ART, to levels nearly in healthy animals. Further, ART also showed recovery effects for $\beta$-diversity, which reduced the increased level of $\beta$-diversity due to SIV infection to a level comparable to the healthy control (Fig. 1C). These results demonstrate that overall ART is beneficial in reversing the abnormality of gut microbiome diversity caused by SIV infection.

In this NHP model of SIV infection, we observed many changes of gut microbiome that similar to HIV infection. For instance, within the phylum *Firmicutes*, the class *Clostridia* is involved in anti-inflammatory roles by producing butyrate and other short-chain fatty acids (SCFA)^33–37^. The reduction of class *Clostridia* in HIV infection was observed in numerous reported studies^16–18,38–42^, and similarly, we found a slight reduction of this class in untreated SIV-infected animals (Fig. 6B). Specifically, within the class *Clostridia,* in comparison to ART1, SIV infection significantly increased pathogenic bacteria families such as *Lachnospiraceae, Clostridium XIVa,* and diminished commensals such as *Ruminococcaceae* and *Butyricicoccus,* consistent with findings in HIV infection^16,38,42–44^. Note that the role of commensal family *Ruminococcaceae* could be either protective or disruptive within the gut microbial community, such as the production of anti-inflammatory SCFA^45^ or the degradation of host mucus and potential pro-inflammatory role in conditions like intestinal bowel disease^46^, suggesting a balanced maintenance of these bacteria species is essential.

Further, consistent with observations found in HIV infection, the phylum *Bacteroidetes* that includes the families of *Prevotellaceae*, *Porphyromonadaceae*, *Bacteroidaceae*, and *Rikenellaceae*, exhibited a more heterogeneous pattern of changes in SIV infection and ART (Fig. 6C). The family *Prevotellaceae* was enriched in SIV-infected animals, which is associated with inflammation, particularly during the chronic phase of inflammatory diseases such as ulcerative colitis^47–49^ and is also associated with activation of gut dendritic cells^50^. Whereas the two families *Bacteroidaceae* (mostly driven by the abundance of the genus *Bacteroides*) and *Rikenellaceae* were reduced during SIV infection*,* which were also found depleted in HIV infection^44,50–52^. *Bacteroidaceae* is commonly considered to play an anti-inflammatory role^53,54^, and *Rikenellaceae* is considered as bile tolerant family^55^ that displays protective properties against C. difficile infection^56^, but may have a positive associations with type1^57^ and type2^58^ diabetes mellitus.

It has been shown that following HIV infection, there is an abundant change of two dominant phyla, the *Firmicutes* and *Bacteroidetes,* with increases of the former and decreases of the latter^16,17,39,41,42,59^. However, in SIV infection, we found a comparable composition of *Firmicutes* between SIV-infected animals and animals on ART, with a slightly increased number of *Bacteroidetes* in SIV-infected animals. Given that the relatively better control of confounding factors in the NHP model, with the same dose, same route of SIV infection, and same ART, the observed increase of *Firmicutes* or decrease of *Bacteroidetes* in humans may be due to more complexed factors and might not be directly caused by HIV infection.

As shown in our study, following 2 months of ART (ART1), SIV-infected animals restored a partial normalization of the microbiome towards the NON_SIV controls with full virus suppression (e.g., Fig. 1), although not all studies found the same trend following ART in SIV or HIV under different conditions or treatment periods^11,35,39,51,60^. In our study, under the phylum *Spirochaetes*, from class Spirochaetae to family Spirochaetaceae and genus *Treponema*, SIV infection led to a certain degree of reduction of these microbial groups (Fig. 6A–D). The genus *Treponema* lineage has been identified as part of rumen GI flora that helps in digestion of pectin, suggesting a possible role in digesting vegetable materials in the GI tract^6,61^. Although the mechanisms of reduction of Treponema in SIV infection and whether it influences the diet digestion are unclear, ART was able to reverse this lineage back to the level very close to that observed in the healthy animals.

It is noteworthy that short-term ART may cause some adverse effects such as significant increase of phylum *Proteobacteria* shown in SIV-infected pig-tailed macaques with initiation of ART^11^ and also observed here with 2 months of ART (Figs. 2B, 3) and 6 months of ART (Fig. 7A). The increase of *Proteobacteria* was also shown in primary SIV infection^62^ and HIV-1 infection^18,40,50,59^, which might induce proteobacteria to preferentially translocate to the peripheral system. In addition, the enrichment of *Proteobacteria was* correlated with the stimulation of kynurenine pathway of tryptophan metabolism^18^ and was associated with chronic inflammation, T-cell activation and serum markers of innate immune activation that was found to trigger obesity, diabetes mellitus, hypertension^17^ etc. Many HIV patients gain weight after ART initiation, and the microbiome changes after ART initiation to weight gain is an important area for ART-caused cormobidity study^63^. Nevertheless, in this study we observed that with longer therapy, *Proteobacteria* was reduced back to normal level when treated for 9 months (Fig. 7A), indicating that a longer therapy could prevent this phylum’s translocation and potentially reduce systemic immune activation.

We also found other beneficial changes of bacterial microbiome by longer ART. Specifically, the abundance of helicobacter (under the genera of *Helicobacteraceae*) was dramatically reduced after 6 months of ART (ART2) and further reduced after 9 months of ART (ART3) (Fig. 7B). Helicobacter is a specific *Proteobacteria* genera that have been involved in pathogenic activities, which are related to stomach infections including chronic gastritis, peptic ulcer disease, gastric cancer^64,65^, and the co-infection of *Helicobacter* is also associated with HIV disease progression^66,67^. Moreover, genera *Clostridium XIVa* (OTU00023) and the *Clostridiales_unclassified* (OTU00139) in the families of Lachnospiraceae and Clostridiales under the phylum of *Firmicutes* were significantly increased after longer ART (9 months of ART) but not 2 months or 6 months of ART (Fig. 7C,D). They are indispensable regulators of intestinal homeostasis^68^. Meanwhile, the genera *Coprococcus* (OTU00109) and *Ruminococcus* (OTU00156) were also markedly increased at 9 months of ART (Fig. 7E,F), both of which play an anti-inflammatory role by producing butyric acid and SCFA in humans.

The alteration of the gut microbiome and leaky gut can cause microbial translocation that leads to systemic immune activation^8,29,69^, and systemic microbial translocation may be associated with the level of anti-CD4 autoantibody production in HIV-infected patients with ART^70^. Here we also found SIV infection caused higher levels of bacterial LPS in plasma, a marker of microbial translocation. Accumulation of LPS during HIV or SIV stimulates activation of monocytes or macrophages, of which, the presence could be inferred by secretions of sCD14. Although sCD14 was not significantly increased in the SIV+ group in our study (Fig. 8B), the trend of higher sCD14 and significantly higher LPS suggest the increased leakiness of microbial products which could cause systemic immune activation. Therapies that target the metabolic activity and composition of the gut microbiome alterations due to HIV/SIV infection could be beneficial in the stage of ART.

One should mention that there were some limitations of our study. Firstly, the small sample size of our longitudinal analysis of ART limited the power of the study. Secondly, our results relied on fecal microbiota for inference and characterization of the intestinal microbial community signature. It may be desirable to conduct further metagenomic analysis on mucosal samples to profile mucosa-related microbial community. Thirdly, we did not directly assess the microbial translocation and further research on direct evidence of microbial translocation or immune activation may be performed.

Altogether, this study revealed that gut dysbiosis with a change in bacterial diversity and taxonomic composition, compared with Non_SIV animals, was induced by SIV infection. The fecal bacterial microbiota upon ART appeared to recover towards the direction of normal NON_SIV controls. Thus, ART may benefit SIV+ animals by enhancing the microbiome diversity that was compromised by SIV infection. Moreover, the gut bacterial community maintained dynamic changes during the time course of ART, and longer ART may be beneficial to restore some of gut microbiota to healthy levels. These results highlight the need for further research to understand the interactions of ART drugs with gut microbial communities and their pharmacokinetics. Overall, gut microbiome profile represents another dimension of HIV/SIV pathophysiology, and treatments that regulate gut microbiota in HIV patients may potentially balance gut microenvironment, repair the gut damage, and control residual inflammation in individuals receiving long-term ART.

## Materials and methods

### Animals and SIV infection

The Indian rhesus macaques were housed at the Tulane National Primate Research Center (TNPRC), which is fully, AAALAC accredited. All procedures were IACUC-approved and were performed in accordance with the Guide for the Care and Use of Laboratory Animals. The animal housing rooms were maintained on a 12:12-h light:dark cycle, with a relative humidity of 30–70% and a temperature of 64–84 °F (18–29 °C). All animals were fed a standard, commercially formulated NHP diet with fruit offered at least three times weekly, as part of the enrichment program. A total of 25 animals were utilized in this study, which included healthy controls (n = 14), SIV infected (n = 9) and ART treated (n = 4). Indian rhesus macaques were inoculated intravenously with 100 TCID50 of SIVmac239 6 weeks before ART. The virus stock of SIVmac239 was collected by culturing from CEMx174 cells and provided by the production core of TNPRC^71^.

### Antiretroviral therapy (ART)

After 6 weeks of SIV infection, animals were treated with ART, which included nucleoside reverse transcriptase inhibitors (R)-9-(2-Phosphonomethoxypropyl) adenine (PMPA, tenofovir; 20 mg/kg) and beta-2′,3′dideoxy-3′-thia-5-fluorocytindine (FTC, Emtricitabine; 40 mg/kg) daily by subcutaneous injection, and an integrase inhibitor raltegravir (20 mg/kg) orally BID for up to 9 months. For the first part of the study samples were collected upon treatment of ART after 2 months (ART1) then for longitudinal analysis additional samples were collected after 6 months (ART2), and 9 months of ART (ART3).

### Plasma and fecal sample collection

Plasma samples were obtained from 10 ml EDTA-blood by centrifuged at 1500 rpm for 10 min to separate the plasma from the cells. Approximately 5 g fresh fecal sample was collected in a sterile plastic cup from each animal and stored at − 80 °C until use for genomic DNA extraction, from control animals and in animals on ART longitudinally during SIV infection and ART. A total of 14 fecal samples were collected from healthy animals, seven from chronical SIV-infected rhesus macaques, and a total of 12 longitudinal samples collected during ART at 2 months (ART1), 6 months (ART2), and 9 months (ART3) from 4 SIV-infected treated rhesus macaques when plasma viral loads started to decline, became undetectable, and maintained steady, undetectable levels, respectively.

### Quantification of SIV viral RNA in plasma

Viral RNA was extracted from plasma samples by using High Pure Viral RNA kit (Roche, Indianapolis, IN, USA) with minor modification. SIV RNA levels were monitored by real-time quantitative PCR (qPCR) assay by the Pathogen Detection and Quantification Core of Tulane National Primate Research Center as described elsewhere^72^. Primers and probe were designed from the conserved gag region, which covered the detection of SIVmac239, SIVmac251 and SHIV viruses. The cut-off threshold for detection was 80 copies/ml (Suppl. Table S1).

### Biomarkers for microbial translocation

Pierc LAL Chromogenic Endotoxin Quantitation was performed to quantify gut microbial lipopolysaccharide (LPS) (Thermo Fisher Scientific, Waltham, MA, USA) according to the manufacture’s instruction. A small volume of the sample (10 μL) was combined with the Limulus Amebocyte Lysate, and endotoxins in the sample activate the proteolytic activity of Factor C. The chromogenic substrate was added, and the activated protease catalyzed the cleavage of p-nitroalinine (pNA), which produced yellow color that can be quantitated by measuring the absorbance at 405 nm (A405) and extrapolating against a standard curve.

Enzyme-linked immunosorbent assay (ELISAs) was also performed to quantify plasma marker for microbial translocation, including soluble CD14 (sCD14) (RayBiotech, Peachtree Corners, GA, USA) according to the manufacture’s protocol. Briefly, all reagents, samples and standards were prepared as instructed in the manual. Standard or a sample (100 µl) was added to each well and incubated 2.5 h at RT. Biotin antibody (100 µl) was added to each well and incubated 1 h at RT. Then, 100 µl of prepared streptavidin solution was added to each well and incubated for 45 min at RT. This step was followed by the addition of 3,3′-5,5′tetramethylbenzidine TMB One-Step Substrate Reagent for 30 min. The reaction was stopped by adding 50 µl of stop solution and read at 450 nm immediately.

### Extraction and purification of genomic DNA

Total DNA was extracted from 180 to 220 mg stool sample using the QIAamp DNA stool mini kit (Qiagen, Inc. Valencia, CA) following the manufacturer’s protocol for pathogen detection. The samples were lysed in lysis buffer then centrifuged and supernatant was collected. The collected supernatant treated with protease to digest the proteins, the digestion samples were added on binding column then proceed with the washing steps, the washing DNA was eluted. The amount of DNA was determined using a NanoDrop. All extracted DNA was stored at − 80 °C before further analysis.

### DNA library construction and 16S rRNA gene sequencing

Samples from each animal and at each time point were used to establish 16S rRNA gene libraries. The V3–V4 region of 16S rRNA gene was PCR amplified with the universal primers 338F (5′–3′) and 806R (5′–3′) to generate an amplicon about 469 bp in length. Each PCR product was purified and checked on 0.8% agarose gel. Sequencing was performed using the MiSeq platform (according to the manufacturer’s specifications) to generate paired-end reads of 300 base lengths in each direction.

### Sequencing data analysis and statistical analysis

All reads were demultiplexed, preprocessed, and subsequently analyzed with the MiSeq SOP of Mothur (https://mothur.org/wiki/MiSeq_SOP)^73^. Briefly, the SOP includes step-wise data cleaning procedures of reducing sequencing and PCR errors, removing duplicate sequences, aligning the data to reference database (using the file silva.bacteria.fasta), filtering the sequences to remove the overhangs at both ends, removing the chimeric sequences, etc. The final number of sequences remained after the above data cleaning procedures is 1,110,191. Based on the cleaned data, we clustered sequences into OTUs using “dist.seqs” and “cluster” functions. We then used “phylotype” command to bin the sequences into phylotypes according to their taxonomic classification.

Statistical analyses on the initial datasets generated with Mothur^74^ were conducted using the pipeline of microbiome R package (https://microbiome.github.io/microbiome)^75^ to identify and compare the composition and abundance of microbiome in the gut among treatment groups. Other R packages used in the pipeline include phyloseq^35,36^ and tidyr.

Specifically, we performed global indices analysis using the “global” function to compare richness, evenness, diversities, dominance and rarity among the three groups (ART, NON_SIV, and SIV-infected).

For identification of OTUs with differential abundance across different treatment groups, we filtered the data to keep only “core” OTUs by setting the detection threshold at 0.01 to alleviate the multiple testing problem. We then used non-parametric ANOVA test (Kruskal. Test function) to compare the OTUs among three treatment groups or across different time points. Using the core OTUs as input, we performed LEfSe (linear discriminant analysis (LDA) effect size) analysis^24^ to identify bacteria species with differential abundance.

### Disclaimer

The content is solely the responsibility of the authors and does not necessarily represent the official views of the National Institutes of Health.

## Supplementary information

Supplementary Tables.

## Data Availability

*Accession codes* The data are available at the NCBI Sequence Read Archive (SRA) under accession no. PRJNA665255 (https://www.ncbi.nlm.nih.gov/sra).
