# Supplementary material for: Alterations of the gut bacterial microbiota in rhesus macaques with SIV infection and on short- or long-term antiretroviral therapy
Source: Sci Rep. 2020 Nov 4;10:19056. doi: 10.1038/s41598-020-76145-8 (PMC7642356; doi:10.1038/s41598-020-76145-8)
Supplement: Supplementary file 1 — Supplementary Tables. [file 41598_2020_76145_MOESM1_ESM.pdf]

Alterations of the gut bacterial microbiota in rhesus macaques with SIV infection and on short- or long-term antiretroviral therapy

Summer Siddiqui[1], Duran Bao[2], Lara Doyle-Meyers[1], Jason Dufour[1], Yuntao Wu[3], Yao-Zhong Liu[2], Binhua Ling[1,4,5*#]

[1]Tulane National Primate Research Center, Covington, LA 70433

[2]Department of Biostatistics and Data Science, School of Public Health and Tropical Medicine, Tulane University, New Orleans, LA 70112

[3]National Center for Biodefense and Infectious Diseases, Department of Molecular and Microbiology, George Mason University, Manassas, VA 20110

[4]Tulane Center for Aging, School of Medicine, Tulane University, New Orleans, LA 70112

[5]Department of Microbiology and Immunology, School of Medicine, Tulane University, New Orleans, LA 70112

***Reprints or correspondence, and current address:**

*Dr. Binhua Ling

# Current address: Texas Biomedical Research Institute

8715 W Military Dr, San Antonio, TX 78227

Phone: (210) 258-9186

E-mail: bling@txbiomed.org

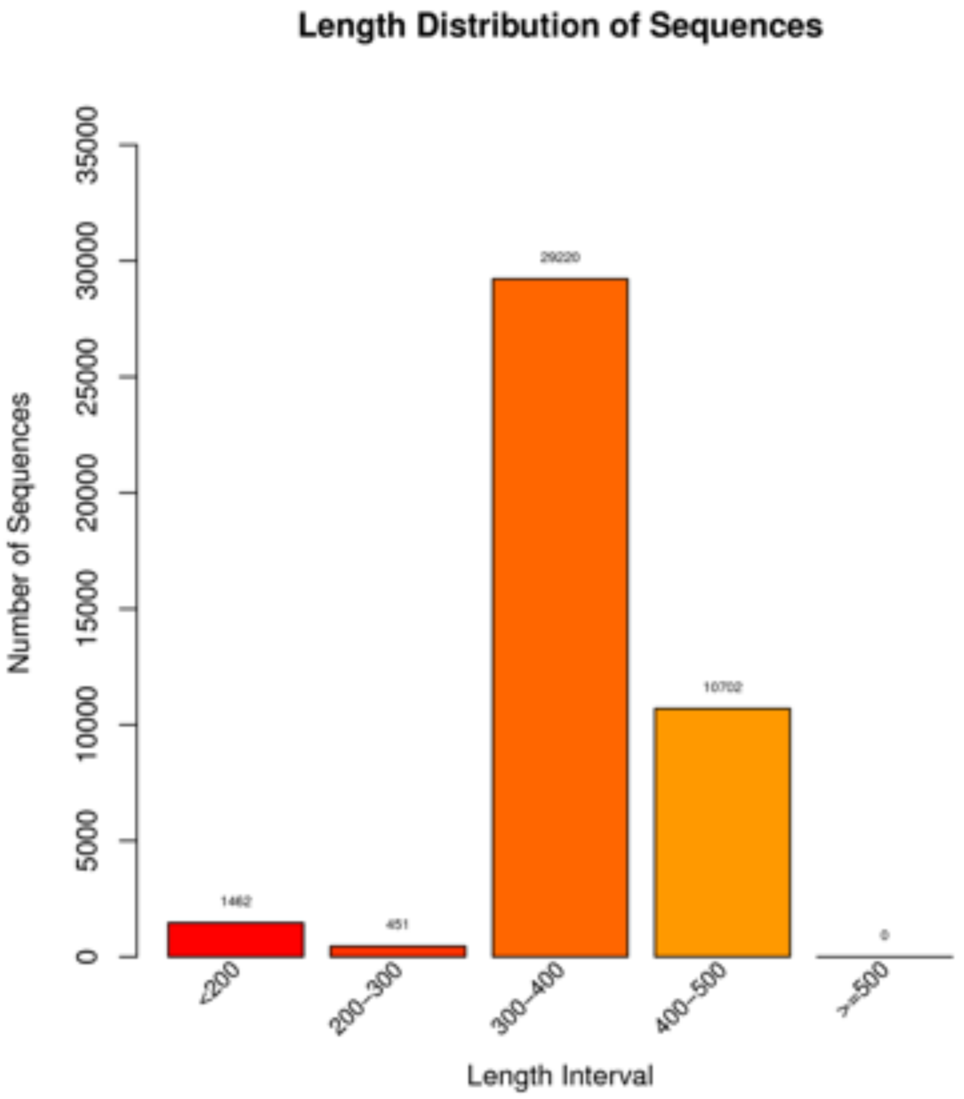

Supplementary Figure S1: Length of distribution of sequences.

The figure illustrates the length distribution of all the reads from all the samples used in this study. The median length was 469 nt.

Supplemental Table S1. Phylum level taxonomic category identification

Group	Firmicutes (%)	Bacteroidetes (%)	Spirochaetes (%)	Proteobacteria (%)
NON_SIV	57.61	25.98	10.40	2.87
SIV+	52.85	34.60	11.52	2.90
ART1	49.70	26.79	16.30	6.67
ATR2	46.41	28.88	18.70	8.22
ART3	64.40	23.86	10.58	2.36

Supplemental Table S2. List of differential operational taxonomic units between three groups that were treated with different periods of therapy

OTU	Size	Taxonomy
Otu00010	19575	Bacteria(100);"Proteobacteria"(100);"Proteobacteria"_unclassified (100);"Proteobacteria"_unclassified(100);"Proteobacteria"_unclassified(100);"Proteobacteria"_unclassified(100)
Otu00126	2510	Bacteria(100);"Proteobacteria"(100);Epsilonproteobacteria(100);Campylobacterales(100);Helicobacteraceae(100);Helicobacter(100);
Otu00023	12981	Bacteria(100);Firmicutes(100);Clostridia(100);Clostridiales(100);Lachnospiraceae(100);Clostridium_XlVa(81);
Otu00109	3023	Bacteria(100);Firmicutes(100);Clostridia(100);Clostridiales(100);Lachnospiraceae(100);Coprococcus(100);
Otu00156	1905	Bacteria(100);Firmicutes(100);Clostridia(100);Clostridiales(100);Ruminococcaceae(100);Ruminococcus(96);

Note: These OTUs were selected based on the standard *p* value <0.05 among groups of ART1, ART2, and ART3.

Supplemental Table S3 : Primary information of SIV-infected rhesus macaques on antiretroviral therapy

Animal	Sex	Route of infection	Length of infection (weeks)	Pre-ART PVL (copies/ml)	Duration of ART (months)	PVL after 2 months of ART (copies/ml)	PVL after 6 months of ART (copies/ml)	PVL at the end of ART (copies/ml)
KC81	F	I.V.	6	1.69×10^{6}	2 ~ 9	1.02×10^{3}	2.10×10^{2}	UD
KP09	M	I.V.	6	3.46×10^{6}	2 ~ 9	5.96×10^{2}	5.0×10^{1}	UD
LB29	M	I.V.	6	3.12×10^{6}	2 ~ 9	3.24×10^{2}	1.87×10^{2}	UD
LE61	F	I.V.	6	4.17×10^{6}	2 ~ 9	5.00×10^{1}	5.00×10^{1}	UD

I.V. : Intravenously

PVL: Plasma viral load

UD: Under the limit of detection
